# Alexander P. Turner, PhD (1940–2016)

**DOI:** 10.1120/jacmp.v17i5.6645

**Published:** 2016-09-08

**Authors:** Michael D. Mills

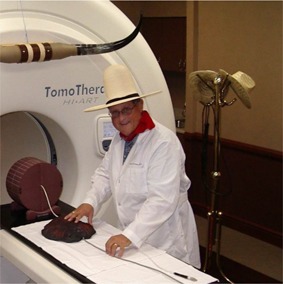

Alex passed away on April 15 from complications due to esophageal cancer with metastasis to the lung and liver. He was 76 years old.

Alex Turner earned a PhD from the University of Oklahoma and began his career in medical physics with his entry as a post‐doctoral fellow at the University of Texas MD Anderson Hospital in the early 1970s. His contemporaries include Bernie Hranitzky, Walter Grant, Leroy Humphries, Jim Purdy, and George Oliver.

Alex served for a number of years as the physics director of the Central Arkansas Radiation Therapy Center (CARTI) in Little Rock, AR. Later he served as the director of physics in various locations, including Sacramento (CA), Oklahoma City (OK), Fort Worth (TX), and eventually settled in retirement in Breckenridge, CO. His last position of which I am aware was for the San Jose, California‐based Xoft Corporation in service to their electronic brachytherapy product.

He served as Chair of the American College of Medical Physics (ACMP) in 1997, and was a Fellow of both the ACMP and the AAPM.

Alex, who loved skiing, was among a small group of physicists that founded what eventually became the Winter Institute of Medical Physics (WIMP). No doubt, his love of the mountains persuaded him to retire at high altitude in Colorado.

Alex had many talents, first and primarily as a clinical medical physicist. He was one of the first wave who acquired the Nomos Peacock system and was a pioneer in intensity‐modulated radiation therapy (IMRT). In addition, he excelled in managing and directing large projects. He was also an inventor. I remember commissioning and using a rotating half‐beam block with half‐moon‐shaped wedges that he developed. This gadget worked beautifully in the simpler era that was the late 1980s.

My primary interaction with Alex occurred at the birth of the *JACMP*. In November of 1997, I approached Alex, who was at the time Chair of the ACMP. He supported the concept of an open‐access journal for clinical medical physics publications, and served on the original ACMP Task Group for a Journal of Clinical Physics. The *JACMP* likely would not have come into existence without Alex's active and enthusiastic support.

For all of Alex Turner's accomplishments and creativity in medical physics, those of us who knew him know that his real passion for living stemmed from his relationships with people, especially students and young physicists, and his love of the outdoors, particularly hiking and skiing. He leaves behind his beloved wife, Karen, and his two sons, John with his wife Jennifer, and Michael with his spouse Claire. Alex and Karen have five grandsons who were the joy of Alex's life. Alex was also well‐known internationally, with friends throughout the world.

Alex's efforts in promoting the value of the medical physics profession are well known, and will be long remembered. He never missed the opportunity to educate a student or new professional. He was not prone to lecture, but rather his way was to freely share what knowledge he had. Sometimes that knowledge was technical, sometimes political or professional, and sometimes even personal. Also, he let his personal philosophy invade his work persona; his integrity, personable nature, and attitude characterized his work presence. He also knew how to separate work from his personal life and focus on life outside of physics when that's where he was. This was the way he conducted himself throughout his career, always being a man of high principle and always a true gentleman. Alex truly was a credit to our profession. He will be greatly missed.

## COPYRIGHT

This work is licensed under a Creative Commons Attribution 3.0 Unported License.

